# Microplastic Munchies: Exploring Microplastic Trophic Transfer Potential Between Two Key Prey Fish Species and Resident Common Bottlenose Dolphins ( *Tursiops truncatus* ) in Sarasota Bay, Florida

**DOI:** 10.1111/mms.70198

**Published:** 2026-05-15

**Authors:** Estella Martin, Randall S. Wells, Elizabeth J. Berens McCabe, Miranda K. Dziobak, Savannah M. Case, Tita Curtin, Eric Conger, Ayushi Gaur, Millie A. Knowles, Robyn Faulkner Allen, Christina N. Toms, John E. Weinstein, Leslie B. Hart

**Affiliations:** ^1^ Department of Public Health Sciences and Administration School of Health Sciences, College of Charleston Charleston South Carolina USA; ^2^ Sarasota Dolphin Research Program Brookfield Zoo Chicago, ℅ Mote Marine Laboratory Sarasota Florida USA; ^3^ Department of Environmental Health Sciences, Arnold School of Public Health University of South Carolina Columbia South Carolina USA; ^4^ Rocky Vista University Montana College of Osteopathic Medicine Billings Montana USA; ^5^ Department of Biology School of Natural and Environmental Sciences, College of Charleston Charleston South Carolina USA; ^6^ Department of Psychology New College of Florida Sarasota Florida USA; ^7^ Department of Biology The Citadel, Charleston Charleston South Carolina USA

**Keywords:** cetacean, contaminant, food web, marine mammal, plastic pollution

## Abstract

Microplastics have been identified in hundreds of species, with evidence of trophic transfer via contaminated prey. Sarasota Bay common bottlenose dolphins ( *Tursiops truncatus* ) serve as sentinels of coastal pollution, including plastics and chemical plasticizers. Previous research confirmed microplastic ingestion in these dolphins (100.0%, *n* = 7) and extensive contamination in 11 common prey species (96.5%, *n* = 86). This study compared microplastic characteristics in dolphins, Gulf toadfish ( *Opsanus beta* , *n* = 30) and pinfish ( *Lagodon rhomboides* , *n* = 35), to assess potential trophic transfer. Dolphin ingestion was evaluated using gastric (*n* = 23) and fecal (*n* = 15) samples from catch‐and‐release health assessments (2022–2024), while prey gastrointestinal and muscle tissues were screened. Particles were prevalent across samples (dolphins: 82.6%, pinfish: 97.1%, toadfish: 96.7%), with fibers as the dominant shape. Raman spectroscopy confirmed plastic polymers (PL, PP, and PET) in all species. Pinfish had a higher median particle load (1.4 particles/g tissue) than toadfish (0.6 particles/g tissue; *p* = 0.006). Based on estimated daily food intake (5.96–6.45 kg) and diet composition (toadfish—34.1%, pinfish—38.1%), Sarasota dolphins may ingest more than 10,000 particles/day from these fish alone. Contaminated prey may be vectors for dolphin microplastic exposure. This study emphasizes the need for further research on microplastic pathways in marine food webs and implications for wildlife.

## 1 Introduction

Ocean microplastic contamination is a worsening issue worldwide. Microplastics are plastic particles that are smaller than 5 mm, which occur in the marine environment as a result of plastic debris breaking down due to natural forces (i.e., wind, ocean currents, waves, sunlight (UV radiation), heat, and some microorganisms; Xi et al. 2022). Microplastics can also be intentionally produced, such as plastic pellets used in manufacturing, cosmetic beads, glitter, and plastic fibers used in synthetic textiles (Rochman 2020; Yurtsever 2019), which can also contaminate the ocean. The microplastic particles afloat at the surface of the world's oceans rose from an estimated 15 to 51 trillion particles in 2015 (van Sebille et al. 2015) to between 82 and 358 trillion (mean = 171 trillion) particles by 2019 (Eriksen et al. 2023), reflecting a five‐fold increase in global marine microplastic contamination in just 5 years.

Recent studies have detected microplastics in a wide variety of marine organisms, including zooplankton (Setälä et al. 2014), seagrasses (Goss et al. 2018), worms (Porter et al. 2023), bivalves (Ding et al. 2019; Kazour et al. 2019), crustaceans (Farrell and Nelson 2013; Gopal et al. 2022; Saborowski et al. 2022), fish (Barboza et al. 2020; Lo et al. 2023; Roch et al. 2020), sea turtles (Caron et al. 2018; Duncan et al. 2019), and marine mammals (Besseling et al. 2015; Courville et al. 2024; Dool and Bosker 2022; Nelms et al. 2018). In marine fish alone, hundreds of species have ingested microplastics (Barboza et al. 2023; Markic et al. 2020). Organisms at a variety of trophic levels may ingest microplastics via purposeful ingestion, accidental ingestion, and consumption of contaminated prey. Purposeful ingestion occurs when an organism, such as zooplankton (Setälä et al. 2014), shrimp (Saborowski et al. 2022), or fish (da Costa et al. 2023; Roch et al. 2020), whose typical prey might be roughly the same size as a microplastic particle, confuses suspended particles for food and directly targets it for consumption. Accidental ingestion occurs when an organism unintentionally takes in particles through its gills or swallows them while attempting to capture or consume natural prey, which has been documented in fish (Li et al. 2021) and indiscriminate filter feeders like bivalves (Kazour et al. 2019), humpback whales (Besseling et al. 2015), and some aquatic worm species (Porter et al. 2023). Lastly, organisms may ingest microplastics through direct consumption of contaminated prey, which has also been documented in a variety of organisms, including zooplankton (Setälä et al. 2014), crabs (Farrell and Nelson 2013), fish (Hasegawa and Nakaoka 2021), and seals (Nelms et al. 2018). While few studies have been able to directly observe marine mammals consuming microplastic‐contaminated prey, many have found evidence suggestive of ingestion via discovery of microplastics in the stomachs, gastrointestinal tracts, and feces of several marine mammal species, including seals (Nelms et al. 2018), whales (Besseling et al. 2015), and dolphins (Dool and Bosker 2022; Hart et al. 2022). Microplastics have been found in other tissues such as blubber, melon, acoustic fat, and lung, suggesting that microplastics may be translocated from the digestive system and transferred to other tissues (Besseling et al. 2015; Merrill et al. 2023). A recent study also found microplastic particles in dolphin exhaled breath, indicating inhalation as an additional exposure route for marine mammals (Dziobak et al. 2024).

The ubiquitous presence of microplastics in all levels of marine food webs is highly concerning because of the health risks they pose to marine organisms. Particularly in organisms that filter feed or consume very small prey/food items (e.g., particulate decaying organic matter or plankton), microplastic ingestion can lead to internal blockages and abrasions (Wright et al. 2013; Xie et al. 2021). Organisms can also experience false satiety and nutritional deficiencies as a result of consuming plastic in place of real prey (Korez et al. 2019; Watts et al. 2015; Welden and Cowie 2016). In addition to these gastrointestinal consequences, studies of microplastic‐exposed marine fish, crustaceans, and isopods suggest associations with oxidative stress (Barboza et al. 2020; Cao et al. 2023; Hsieh et al. 2021; Xie et al. 2021), inflammation (Cao et al. 2023), reproductive toxicity (Vo and Pham 2021), and neurotoxicity (Barboza et al. 2020; Lin et al. 2023; Vo and Pham 2021). The egestion of microplastics after initial ingestion can range from hours to weeks depending on the organism (Santana et al. 2017; Setälä et al. 2014) and may affect how many microplastics can accumulate within an individual at one time and, thus, the extent of their health risk. While the presence of the microplastics alone can be harmful (as with false satiety, internal blockages and abrasions, inflammation, and oxidative stress), studies have also shown that microplastics may leach hazardous chemicals added during manufacturing, such as phthalate plasticizers (Bridson et al. 2023; Cao et al. 2022; Li et al. 2024), flame retardants (Li et al. 2024; Sun et al. 2019), antioxidants (Li et al. 2024), bisphenol A (BPA; Koelmans et al. 2014) and nonylphenol (NP; Koelmans et al. 2014). The surface‐to‐volume ratio of microplastics promotes adsorption of contaminants such as organic chemicals (Liu et al. 2022), heavy metals (Liu et al. 2022; Zuo et al. 2024), and pathogens (Lai et al. 2022; Obusan et al. 2015; Tavşanoğlu et al. 2025; Viršek et al. 2017), which can later desorb following uptake by an organism, ultimately functioning as a vehicle for exposure.

Due to the potential negative health impacts of microplastic ingestion, trophic transfer of microplastics in marine ecosystems is a growing concern. Trophic transfer is the travel of energy or compounds through the food chain (Pouil et al. 2018). Many harmful chemicals have been shown to spread through marine ecosystems via trophic transfer, including metals (Gao et al. 2021; dos Lima et al. 2024) and persistent organic pollutants (Dromard et al. 2018; Kim 2020). While available information is limited, there is also evidence to suggest plastics may transfer up trophic levels (Farrell and Nelson 2013; Nelms et al. 2018; Setälä et al. 2014). Nelms et al. (2018) investigated trophic transfer between seals and their prey fish and found consistent polymer types between the seal scat and prey fish samples, suggesting contamination through diet. This raises concerns for resident common bottlenose dolphins ( *Tursiops truncatus* ) in Sarasota Bay, Florida, who are also apex marine predators that consume whole fish, many of which have been found to contain microplastics (Conger et al. 2024; Hart et al. 2023).

Sarasota Bay is a largely enclosed estuary surrounded by a dense human population and experiences a high amount of boating traffic. Human activity facilitates on‐land plastic waste and plastic debris from boating (Gaylarde et al. 2021; Jaini and Namboothri 2023), fishing, littering, and recreational water activities to enter the environment easily, potentially creating a heightened risk of plastic contamination for local marine wildlife (Cózar et al. 2015; Eriksen et al. 2017). The long‐term resident bottlenose dolphins of Sarasota Bay have been studied for more than 55 years. Over the course of these studies, much has been learned about the biology, behavior, ecology, and health of the dolphins. The unique databases and sampling opportunities available for these animals have facilitated assessments of the threats they face, including exposure to pollutants. Recently, new information about microplastics and plasticizers in these animals and their ecosystems has begun to raise concerns about threats to them. Bottlenose dolphins' diets in Sarasota Bay are known from observations of feeding activities and studies of the stomach contents of stranded dolphins (Barros and Wells 1998; Berens McCabe et al. 2010; Wells et al. 2013). The most recent studies of the prey fish preferences of these dolphins indicate that pinfish (*Lagodon rhomboides*, “Lr”) and Gulf toadfish ( *Opsanus beta* , “Ob”) are among the most frequently consumed species (Berens McCabe et al. 2010; Wells et al. 2013).

This study investigated microplastic concentrations in bottlenose dolphins, pinfish, and Gulf toadfish from Sarasota Bay, with a focus on the potential for trophic transfer by assessing overlap of particle characteristics between the species. This study builds upon preliminary evidence of ingested microplastics in the gastric fluid of seven Sarasota Bay dolphins (samples collected in 2019; Hart et al. 2022) and the detection of particles in their common prey species (Conger et al. 2024; Hart et al. 2023). The objectives were twofold: (1) to compare the characteristics of microplastics in gastric and fecal samples collected during 2022–2024 from Sarasota Bay dolphins with those found in two of the top prey fish species (i.e., pinfish and Gulf toadfish) collected during the same period and (2) to evaluate whether these findings suggest trophic transfer of microplastics from fish to dolphins is feasible.

## 2 Materials and Methods

### 2.1 Fish Collection and Sampling

Sarasota Bay is a shallow (up to roughly 4 m deep) estuary along the central west coast of Florida that borders the Gulf of Mexico. Much of the bay consists of seagrass meadows, and a series of barrier islands separates the bay from the Gulf (Wells 2009). The land surrounding Sarasota Bay is densely populated; according to the US Census Bureau, the combined population of Sarasota and Manatee counties in 2023 was 910,108 (U.S. Census Bureau 2023).

**FIGURE 1:**
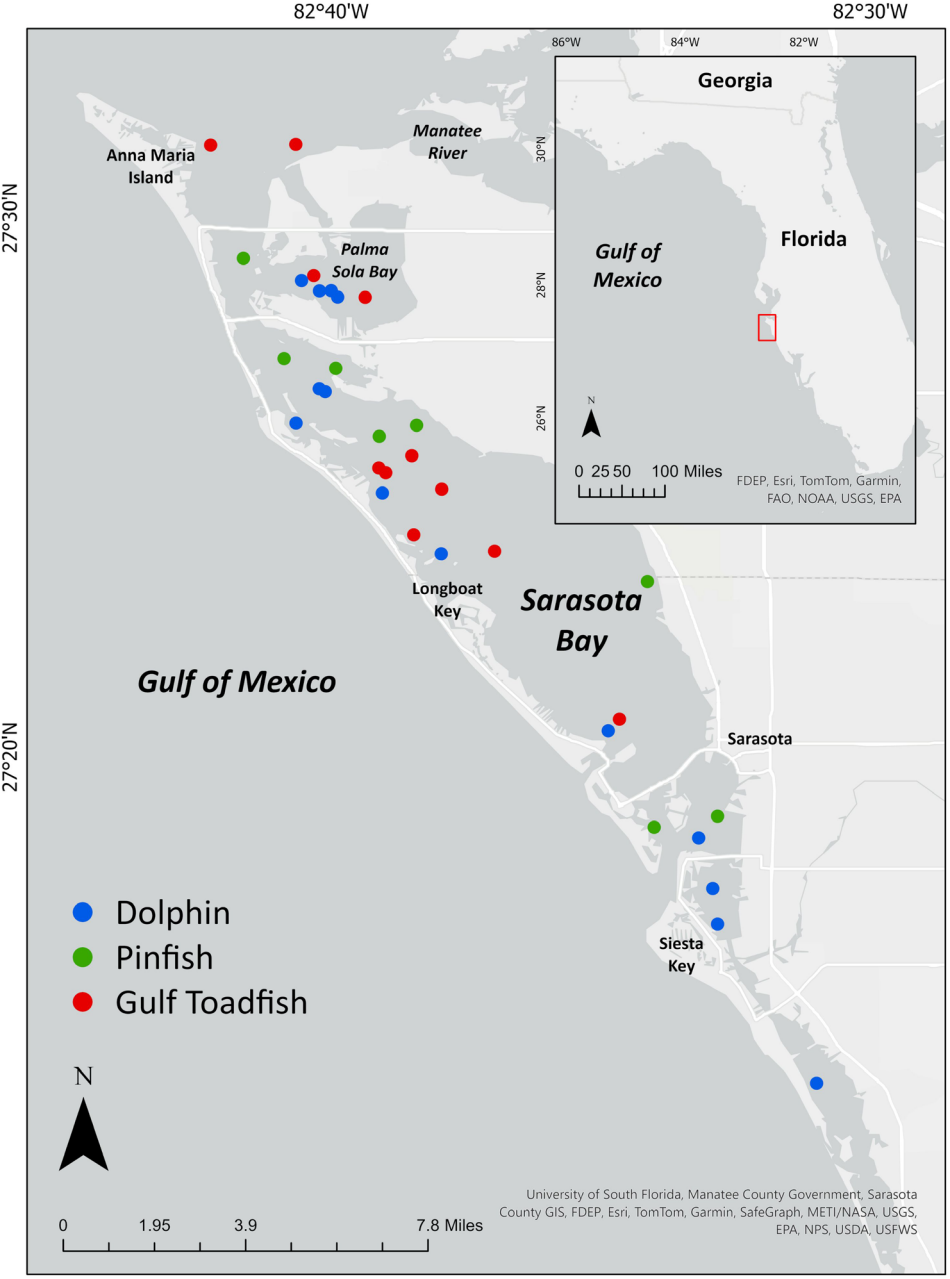
Map of sampling locations of bottlenose dolphins (blue), pinfish (green), and Gulf toadfish (red) in Sarasota Bay, Florida.

Gulf toadfish and pinfish specimens were caught at multiple sites throughout Sarasota Bay (Figure 1) during routine seasonal fish abundance surveys conducted by the Brookfield Zoo Chicago's Sarasota Dolphin Research Program (SDRP; Gannon et al. 2009). Fish collection was done with state‐approved licensing from the Florida Fish and Wildlife Conservation Commission (special activity license nos. 19‐0809A‐SR and 22‐0809‐SR) and approved protocols from the Mote Marine Laboratory's Institutional Animal Care and Use Committee (IACUC). Fish were wrapped in aluminum foil and stored frozen (−20°C) until dissection. Methods to remove muscle and gastrointestinal tissues were previously described (Conger et al. 2024; Hart et al. 2023). Briefly, fish were thawed and removed from the foil before dissections, and the total fish length (cm) and mass (g) were recorded. Each fish was placed on a metal tray, and stainless‐steel forceps and scalpels were used to remove the entire muscle filet from one side, along with the gastrointestinal tract and contents (“gastrointestinal or GI tissue”). Tissue masses were collected prior to storage, and all samples were placed in glass jars before freezing (−20°C) until processing.

### 2.2 Dolphin Sample Collection

Bottlenose dolphin gastric samples were obtained from routine catch‐and‐release health assessments (Barratclough et al. 2019; Wells et al. 2004) led by SDRP during 2022–2024 in Sarasota Bay (Figure 1). Described briefly, dolphins in shallow water were carefully encircled with a seine net and temporarily restrained. Dolphins were identified from distinctive patterns of nicks and notches on the trailing edge of their dorsal fins, or from freeze‐brands applied during previous health assessments (Wells 2018), and an examination of each dolphin's genital slit confirmed their sex (Wells et al. 2025). As most of the dolphins assessed had been monitored regularly from birth, the age could be known as soon as an individual was identified (Wells 2009). For dolphins with unknown ages, an estimate was calculated using dental radiography, bone flipper fusion radiography, and/or epigenetics (Wells et al. 2025). Dolphins were brought aboard a specialized vessel with a padded deck and shading for physical examination and sample collection by expert veterinarians. Gastric samples were collected by passing a small veterinary feeding tube through the esophagus and into the stomach (Goldman et al. 2002; Twiner et al. 2011) and creating negative pressure to draw the fluid up into the tube (Dunshea et al. 2013; Mitchell et al. 2008). Fecal samples were collected opportunistically following defecation on the sampling platform or via suction fecal tube (Goertz et al. 2021; Obusan et al. 2015). All gastric and fecal samples were stored in glass jars and refrigerated prior to microplastic analysis. Sarasota Bay bottlenose dolphin health assessments were conducted using IACUC‐approved protocols (Mote Marine Laboratory approvals 22‐09‐RW2, 23‐09‐RW2) and by permit from the National Oceanic and Atmospheric Administration's (NOAA) National Marine Fisheries Service (NMFS) (Scientific Research Permits #20455 and #26622).

### 2.3 Sample Processing and Microplastic Screening

**FIGURE 2:**
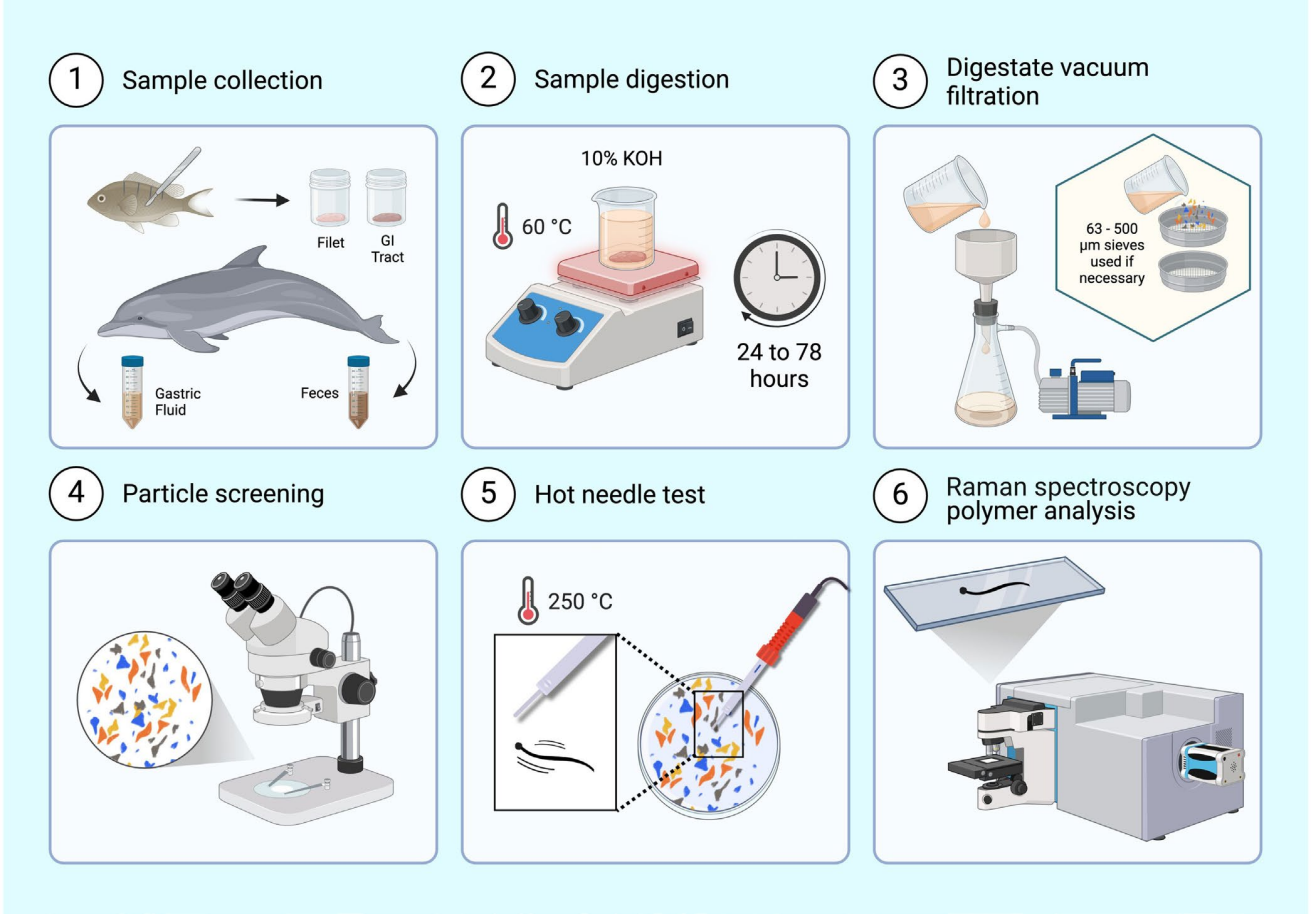
Sample collection, processing, and analysis. Created in BioRender. Martin (2025) https://BioRender.com/q1t8979 and Canva.com.

All fish and dolphin samples were prepared for microplastic screening using a digestion and filtration process (Karami et al. 2017). A 10% potassium hydroxide (KOH) solution (Geppner et al. 2023) was prepared and vacuum‐filtered through GF/A 1.6 μm glass fiber filters to remove any excess particles that remained undissolved or may have landed in the solution during its preparation, thus reducing the likelihood of outside microplastic contamination. Samples were transferred to glass beakers, and the 10% KOH solution was added to each sample to digest organic (non‐plastic) material. Samples were incubated at 60°C under a fume hood for 24 to 72 h. After digestion, samples were vacuum‐filtered onto GF/A 1.6 μm glass fiber filters under a fume hood. For samples particularly congested with solids and organic material, the digestate was filtered through sieves ranging from 63 to 500 μm. The contents held in each sieve were then individually rinsed onto glass fiber filters using filtered water with the vacuum active. All sample filters were transferred to glass Petri dishes, covered, and left to dry (Figure 2).

Once dry, each sample was visually screened for microplastics using a dissection microscope. Suspected plastic particles were characterized by shape (e.g., fiber or fragment; de Piñon‐Colin et al. 2020), texture (e.g., smooth, jagged, compressible, rigid, porous), and color (e.g., transparent, white, black, blue; Shim et al. 2017). The parameters used to identify potential plastic particles were based on descriptions from previous studies. Fibers were characterized by a smooth surface, a longer length than width, and a uniform thickness throughout (Lusher et al. 2020; Mohamed Nor and Obbard 2014). Masses of at least 20 fibers that were tangled to the point where they could not be separated without breakage (Rochman et al. 2019) were classified as fiber bundles. Fragments included any particle shape that could not be classified as a fiber or fiber bundle. Fragments included rigid, irregular shards (Lusher et al. 2020), tire wear particles (TWPs; Leads and Weinstein 2019), films (Hartmann et al. 2019; Lusher et al. 2020), and foams (Hartmann et al. 2019; Lusher et al. 2020; Manikanda Bharath et al. 2023). TWPs were matte black fragments with a cylindrical shape or shredded edges (Leads and Weinstein 2019). Suspected films were indicated by thin, flat particles consisting of only a single layer (Hartmann et al. 2019; Lusher et al. 2020). Foams were compressible, cloud‐like particles with porous, honeycomb‐like surface textures (Hartmann et al. 2019; Lusher et al. 2020; Manikanda Bharath et al. 2023). Suspected plastic particles were distinguished from any remaining non‐plastic particles (e.g., cotton, minerals) by testing them with a hot soldering needle set to 250°C, a temperature at which most polymers melt (Devriese et al. 2015; Lusher et al. 2017; De Witte et al. 2014). Particles were suspected to be plastic if the hot needle caused the particle to bend, shrink, or melt (Figure 2).

A subset of suspected microplastic particles found in dolphin and fish samples was analyzed by Raman microspectroscopy (Xplora Plus with LabSpec 6 software version 6.5, Horiba Scientific) to determine polymer composition (Ertel et al. 2023). These particles were selected based on the most common particle types found among all related samples. Spectra were obtained using a 50× or 100× objective lens with a 785 or 532 nm laser at 0.1% to 100% power, confocal slit width of 100, 300, or 500 μm, hole diameter of 100, 300, or 500 μm with gratings of 600, 1200, or 1800 grooves/mm and varied acquisition times ranging from 0.5 to 90 s (Figure 2). Raman spectra were processed using LabSpec6, and OpenSpecy (Cowger et al. 2021) was used to identify particle matches to spectra of known substances. Only polymer matches with a Pearson r coefficient of at least 0.70 in OpenSpecy were included (Cowger et al. 2021; Dziobak et al. 2024).

### 2.4 Quality Assurance/Quality Control (QA/QC)

Precautions were taken to account for potential field and procedural contamination and to ensure the quality and accuracy of the results. Field blanks were collected by running filtered water through the gastric tube, collection vials, and the fecal sampling apparatus to account for particles that could be associated with sampling equipment (Lusher et al. 2017). For all laboratory procedures, a 100% cotton lab coat and nitrile gloves were worn, and all containers, Petri dishes, dissection trays, and instruments were thrice rinsed with filtered Milli‐Q deionized (DI) water before use (Hart et al. 2023; Lusher et al. 2017; Rochman et al. 2019). During the digestion and filtration processes, all beakers, flasks, and sieves that contained samples or KOH solution and were not in active use were covered with aluminum foil to prevent the deposition of any airborne particles. Procedural blanks were collected during digestion and filtration processes to account for any contamination that could be introduced during laboratory practices (Lusher et al. 2017). Additionally, an empty petri dish was left open on the lab bench during fish dissections to account for ambient particles (Lusher et al. 2017; Rochman et al. 2019). All field, dissection, and procedural blanks underwent the same analysis procedures as the samples (Figure 2). To evaluate the efficiency of microplastic recovery, three positive control samples consisting of commercially purchased polyester, polyethylene, and polystyrene microplastic particles were introduced into sample digestion batches (Rochman et al. 2019). For fish samples, mean recovery percentages were above 70% for all particle types: 73% for foams, 90% for films, and 97% for fibers. For dolphin samples, mean recovery percentages were 87% for foams, 90% for films, and 90% for fibers (Conger et al. 2024; Hart et al. 2023, 2022).

### 2.5 Data Analysis

All statistical analyses were performed using R software v.4.3.2 (R Foundation for Statistical Computing, Vienna, Austria), and statistical significance was evaluated using *α* = 0.05. To account for possible contamination during field collection and laboratory procedures, microplastic data for all sample types (fish muscle tissue, fish gastrointestinal tissue, dolphin gastric fluid, and dolphin feces) were adjusted using particles observed in field, laboratory, and procedural blanks (Conger et al. 2024; Hart et al. 2023). Specifically, any particles found in samples that were also found in blanks were excluded from the results. Descriptive statistics were used to calculate the proportion of dolphin and fish samples containing at least one suspected microplastic particle and to summarize the abundance of particle shapes and colors. For each fish sample, particle abundance (‘particle load’) was calculated as the number of suspected microplastics per gram of tissue (Conger et al. 2024). Particle load between fish species was compared using a Mann–Whitney *U* test. Comparison between tissue types was conducted using a Wilcoxon‐Rank Sum test. Particle load was not calculated for dolphin samples because they were obtained opportunistically, with sample volumes ranging from trace amounts to 40 mL. To evaluate the potential for trophic transfer, particle characteristics (e.g., shape, color, polymer type) were qualitatively compared between fish and dolphin samples. A daily particle ingestion estimate for an adult dolphin consuming pinfish and Gulf toadfish was calculated using the mean particle load for each fish species, diet composition estimates from Wells et al. 2013 (Gulf toadfish—34.1%; pinfish—38.1%), the predicted mass of sexually mature female (149.0 kg; after first birth) and male (161.2 kg) dolphins (Wells et al. 2025), and the percentage of their body mass that dolphins eat each day (4%–5%), summarized by Trites and Spitz (2018).

## 3 Results

### 3.1 Microplastic Ingestion by Bottlenose Dolphins

During health assessments in 2022–2024, gastric (*n* = 23) and fecal (*n* = 15) samples were collected from 23 long‐term resident bottlenose dolphins (Table 1, Figure 1). Slightly more than half of the individuals were male (*n* = 13; 56.5%), and ages among all individuals ranged from 2 to 49 years old. Sample volumes were variable, ranging from trace amounts (< 2 mL) to 40.0 mL (Table 1). When trace volumes of gastric fluid or feces were collected from a dolphin, the collection apparatus was rinsed with filtered water to collect samples for analysis.

**TABLE 1:** Characteristics of bottlenose dolphins sampled for this study during catch‐and‐release health assessments conducted in Sarasota Bay, FL (*n* = 23).

Dolphin ID	Sex	Age (years)	Gastric sample volume collected (mL)	Gastric particles observed	Gastric particle counts	Fecal sample volume collected (mL)	Fecal particles observed	Fecal particle counts
F293	F	3	10.0	Yes	3	—	—	—
F320	M	3	8.2	Yes	2	—	—	—
F322	M	7	30	Yes	76	Trace^a^	Yes	8
F277	F	11	Trace^a^	Yes	4	12.0	Yes	3
F292	M	11	Trace^a^	Yes	12	Trace^a^	Yes	1
F295	F	2	Trace^a^	Yes	4	10.0	Yes	2
F297	F	3	7.0	Yes	1	Trace^a^	No	0
F326	M	4	3.0	Yes	14	10.0	Yes	3
FB07	F	40	17.0	Yes	2	Trace^a^	Yes	3
F137	F	24	30.0	Yes	1	—	—	—
F222	M	25	Trace^a^	Yes	6	—	—	—
F240	M	20	7.0	Yes	6	—	—	—
F261	F	49	10.0	No	0	5.0	No	0
F274	M	14	40.0	No	0	—	—	—
F299	F	6	40.0	Yes	1	5.0	Yes	3
F303	F	4	40.0 (estimated)	Yes	1	2.0	No	0
F305	F	3	6.0	Yes	1	5.0	Yes	2
F328	M	4	Trace^a^	No	0	7.0	No	0
F330	M	5	10.0	Yes	27	2.0	Yes	5
F332	M	4	30.0	No	0	10.0	Yes	5
F334	M	8	19.0	Yes	0	Trace^a^	Yes	1
F336	M	12	Trace^a^	Yes	1	—	—	—
F338	M	19	Trace^a^	No	0	—	—	—

^a^ Trace: < 2 mL sample diluted with filtered water.

Following blank correction, 82.6% of dolphin samples contained at least one microplastic particle. Fibers were the most common particle shape observed across both gastric (65.2%) and fecal (53.3%) samples, followed by several different fragment types; no fiber bundles were observed (Table 2). Although gastric samples generally contained more particles, the proportion of samples with microplastic contamination was similar between gastric (78.3%) and fecal (73.3%) samples (Table 2). A variety of particle colors were identified in both gastric and fecal samples (Figure 4), some of the most common being yellowed, black, transparent, and blue. Particle characteristics from associated field and procedural blanks are available in the data repository.

**TABLE 2:** Counts (total, minimum, and maximum) and proportions of suspected microplastic shapes in gastric (*n* = 23) and fecal (*n* = 15) samples.

Sample type	Particle type	*n*	%	95% CI	Total particles (all samples)	Min–max particles (per sample)
Gastric	Any	18	78.3%	55.8–91.7	172	0–78
	Fibers	15	65.2%	42.8–82.8	126	0–78
	Films (fragment)	3	13.0%	3.4–34.7	10	0–5
	Foams (fragment)	2	8.7%	1.5–29.5	2	0–1
	Irregular shards (fragment)	7	30.4%	14.1–53.0	33	0–27
	TWPs (fragment)	1	4.4%	0.2–24.0	1	0–1
Feces	Any	11	73.3%	44.8–91.1	36	0–8
	Fibers	8	53.3%	27.4–77.7	23	0–5
	Films (fragment)	3	20.0%	5.3–48.6	6	0–4
	Foams (fragment)	3	20.0%	5.8–48.6	3	0–1
	Irregular shards (fragment)	2	13.3%	2.3–41.6	4	0–3
	TWPs (fragment)	0	0.0%	0.0–25.4	0	0

### 3.2 Suspected Microplastics in Bottlenose Dolphin Prey Fish

Whole pinfish (*n* = 35) and Gulf toadfish (*n* = 30; Table 3) were collected from randomly selected fish habitat sites (Berens McCabe et al. 2021) in Sarasota Bay during 2022–2024 (Figure 1). Details on fish collected for this study are provided in Table S1, and suspected particles in a portion of these samples (pinfish *n* = 24; Gulf toadfish *n* = 12) were previously reported in Conger et al. (2024) and Hart et al. (2023). Following blank correction, suspected microplastic particles were present in 97.1% of combined (muscle + gastrointestinal) pinfish samples (*n* = 34) and 96.7% of matched Gulf toadfish samples (*n* = 29; Table 3). Overall, particle load (i.e., number of particles per gram of tissue) was higher in gastrointestinal tissues than muscle (GI vs. muscle median: 2.1 vs. 0.2 particles/g tissue; *V* = 1996, *p* < 0.0001). Between species, particle load was comparable for muscle tissue (median: 0.2 particles/g tissue for both species; *U* = 595.5, *p* = 0.35; Table 3), but significantly higher in pinfish gastrointestinal tissue compared to Gulf toadfish (pinfish median: 4.7 particles/g tissue; Gulf toadfish median: 1.0 particles/g tissue; *U* = 792.0, *p* = 0.0005; Table 3). When combining both tissue types, particle load in pinfish was more than twice that of Gulf toadfish (pinfish median: 1.4 particles/g tissue; Gulf toadfish median: 0.6 particles/g tissue; *U* = 732.5, *p* = 0.006). Particle characteristics observed in dissection and procedural blanks associated with fish samples can be found in the data repository.

**TABLE 3:** Sample mass, particle detection, and particle load (# particles/g tissue) for pinfish and Gulf toadfish.

Fish species	Tissue type	Mass (g), mean (SD)	Number of fish with any particles	% of fish with any particles (95% CI)	Particle load (#/g), mean (SD)	Particle load (#/g), median (IQR)
Pinfish (*n* = 35)	Combined tissues	22.1 (14.5)	34	97.1% (85.1–99.9)	3.1 (4.1)	1.4 (0.6–3.3)
	Muscle	16.0 (13.0)	31	88.6% (73.3–96.8)	0.3 (0.6)	0.2 (0.1–0.3)
	GI	6.1 (3.6)	33	94.3% (80.8–99.3)	11.5 (19.9)	4.7 (1.5–11.7)
Gulf toadfish (*n* = 30)	Combined tissues	18.9 (22.5)	29	96.7% (82.8–99.9)	1.1 (1.4)	0.6 (0.3–1.1)
	Muscle	9.4 (10.4)	20	66.7% (47.2–82.7)	0.2 (0.4)	0.2 (0.0–0.3)
	GI	9.5 (12.4)	28	93.3% (77.9–99.2)	2.3 (3.4)	1.0 (0.3–2.5)

Fibers were the most abundant particle type observed across both fish species, comprising 97.1% of particles in combined pinfish tissues and 83.3% in combined Gulf toadfish tissues (Table 4). Both species also exhibited a variety of particle colors (Figure 4), namely yellowed, white/transparent, black, and blue. Fiber bundles were detected in both species (pinfish combined tissues: 62.9%; Gulf toadfish combined tissues: 23.3%; Table 4). Additional particle types observed in prey fish included irregular shards, foams, and films (Table 4).

**TABLE 4:** Particle attributes for pinfish and Gulf toadfish. Data are reported as counts and percentages of samples with particle shape.

Fish species	Tissue type	Particle type	*n*	%	95% CI
Pinfish (*n* = 35)	Combined tissues	Fiber bundles	22	62.9%	44.9–78.5
		Fibers	34	97.1%	85.1–99.9
		Films (fragment)	23	65.7%	47.8–80.9
		Foams (fragment)	3	8.6%	1.8–23.1
		Irregular shards (fragment)	10	28.6%	14.6–46.3
		TWP (fragment)	10	28.6%	14.6–46.3
	Muscle	Fiber bundles	1	2.9%	0.1–14.9
		Fibers	27	77.1%	59.9–89.6
		Films (fragment)	10	28.6%	14.6–46.3
		Foams (Fragment)	2	5.7%	0.7–19.2
		Irregular shards (fragment)	4	11.4%	3.2–26.7
		TWP (fragment)	4	11.4%	3.2–26.7
	GI	Fiber bundles	22	62.9%	44.9–78.5
		Fibers	30	85.7%	69.7–95.2
		Films (fragment)	19	54.3%	36.7–71.2
		Foams (fragment)	1	2.9%	0.1–14.9
		Irregular shards (fragment)	7	20.0%	8.4–36.9
		TWP (fragment)	6	17.1%	6.6–33.7
Gulf Toadfish (*n* = 30)	Combined tissues	Fiber bundles	7	23.3%	9.9–42.3
		Fibers	25	83.3%	65.3–94.4
		Films (fragment)	10	33.3%	17.3–52.8
		Foams (fragment)	10	33.3%	17.3–52.8
		Irregular shards (fragment)	9	30.0%	14.7–49.4
		TWP (fragment)	5	16.7%	5.6–34.7
	Muscle	Fiber bundles	0	0.0%	0.0–11.6
		Fibers	16	53.3%	34.3–71.7
		Films (fragment)	2	6.7%	0.8–22.1
		Foams (fragment)	1	3.3%	0.1–17.2
		Irregular shards (fragment)	2	6.7%	0.8–22.1
		TWP (fragment)	4	13.3%	3.8–30.7
	GI	Fiber bundles	7	23.3%	9.9–42.3
		Fibers	21	70.0%	50.6–85.3
		Films (fragment)	10	33.3%	17.3–52.8
		Foams (fragment)	9	30.0%	14.7–49.4
		Irregular shards (fragment)	7	23.3%	9.9–42.3
		TWP (fragment)	3	10.0%	2.1–26.5

### 3.3 Particle Characteristic Overlap Across All Three Species

**FIGURE 3:**
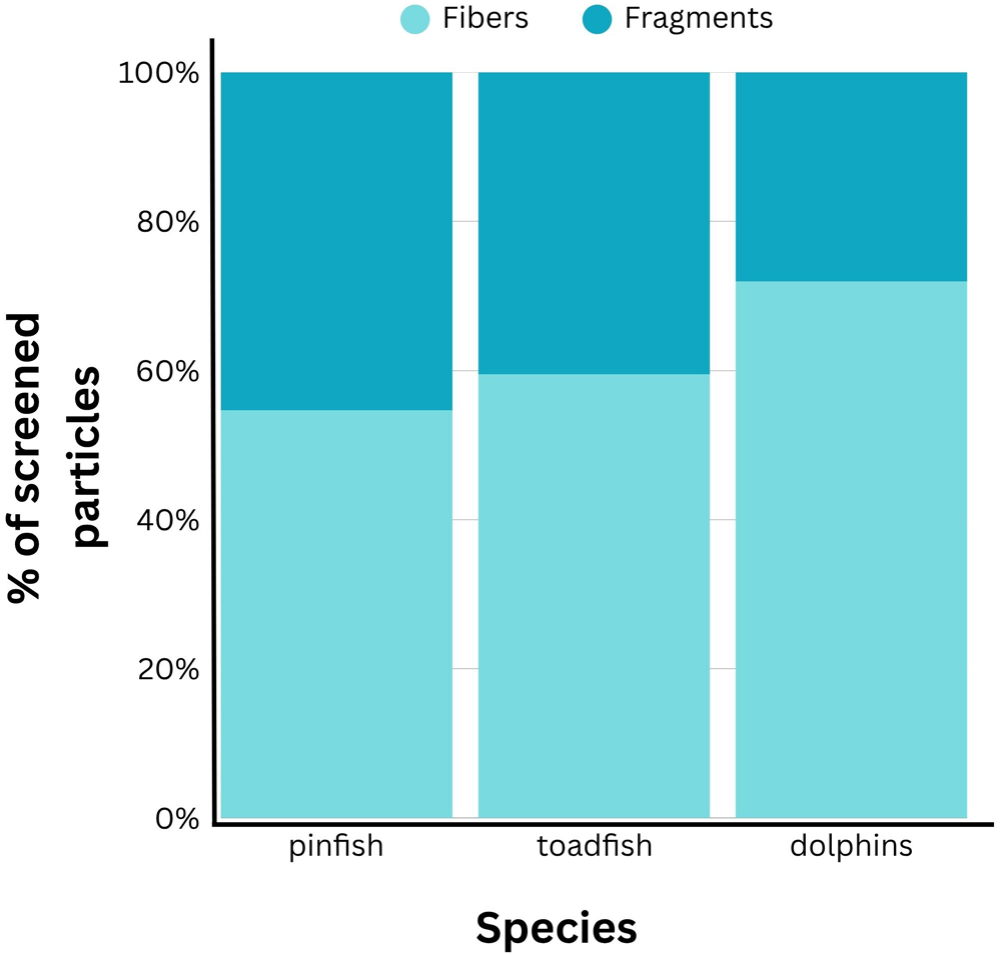
Percentage of fibers and fragments (including films, foams, irregular shards, and tire wear fragments) detected across all pinfish, Gulf toadfish, and dolphin samples. Fiber bundles are not included as they were not detected in any of the three species.

**FIGURE 4:**
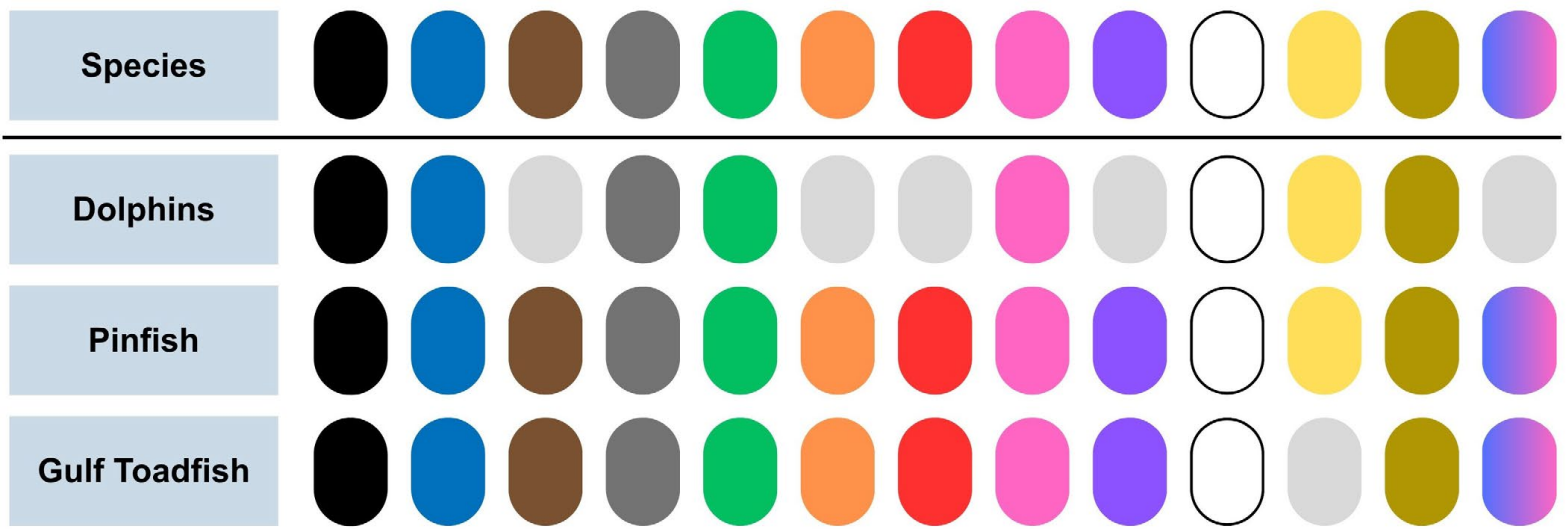
Particle colors found in bottlenose dolphin, pinfish, and Gulf toadfish samples. From left to right the colors are black, blue, brown, gray, green, orange, red, pink, purple, transparent/white, yellow, yellowed, and multi‐colored. Light gray indicates absence.

Across all three species studied, fibers were the most common particle shape detected (Figure 3). A variety of particle colors were present, and all of the colors observed in dolphins were also observed in at least one of the two fish species (Figure 4).

**FIGURE 5:**
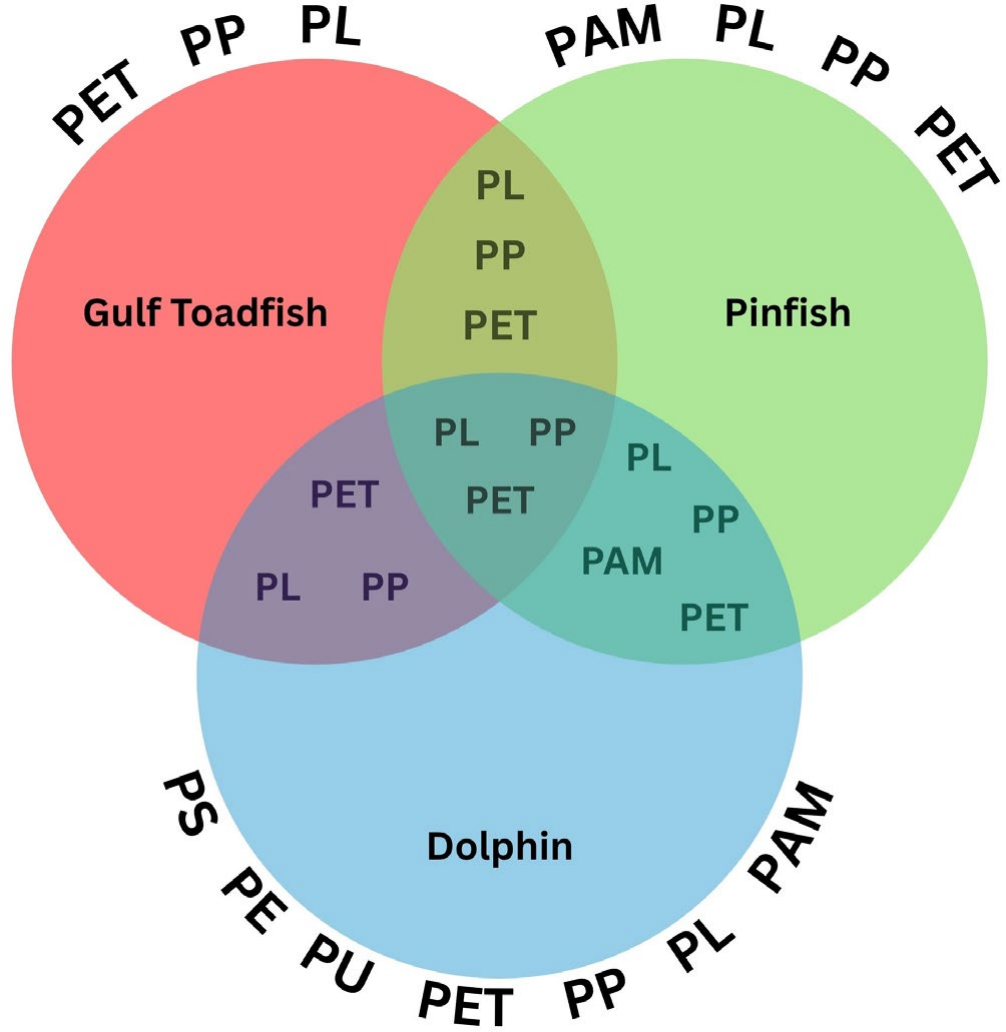
Polymers confirmed among Raman subset particles from bottlenose dolphin, pinfish, and Gulf toadfish samples. Polymer abbreviations: PAM, polyacrylamide; PE, polyethylene; PET, polyethylene terephthalate; PL, polyester; PP, polypropylene; PS, polystyrene; PU, polyurethane.

A subset of particles (*n* = 82) from dolphin, pinfish, and Gulf toadfish samples was analyzed for polymer composition using Raman spectroscopy (Figures 2 and 5). Of the 82 total particles analyzed, 21 (25.6%) were matched to organic materials (e.g., cellulose, cotton, minerals), 27 (32.9%) were inconclusive, and 34 (41.5%) were matched to known plastic polymers (Ertel et al. 2023). Fibers made up the vast majority of organic (mostly cotton) and inconclusive particles. Of the particles matched to plastic polymers, fragments were most common (details about each particle in the Raman analysis subset are available in Table S2). Of the confirmed plastic particles, 10 belonged to pinfish, 9 to Gulf toadfish, and 15 to dolphins. Every polymer present in both fish species was also present in dolphins, including polyester (PL), polypropylene (PP), polyacrylamide (PAM), and polyethylene terephthalate (PET; Figure 5). Dolphins contained the greatest diversity of confirmed polymer types, followed by pinfish (Figure 5).

**FIGURE 6:**
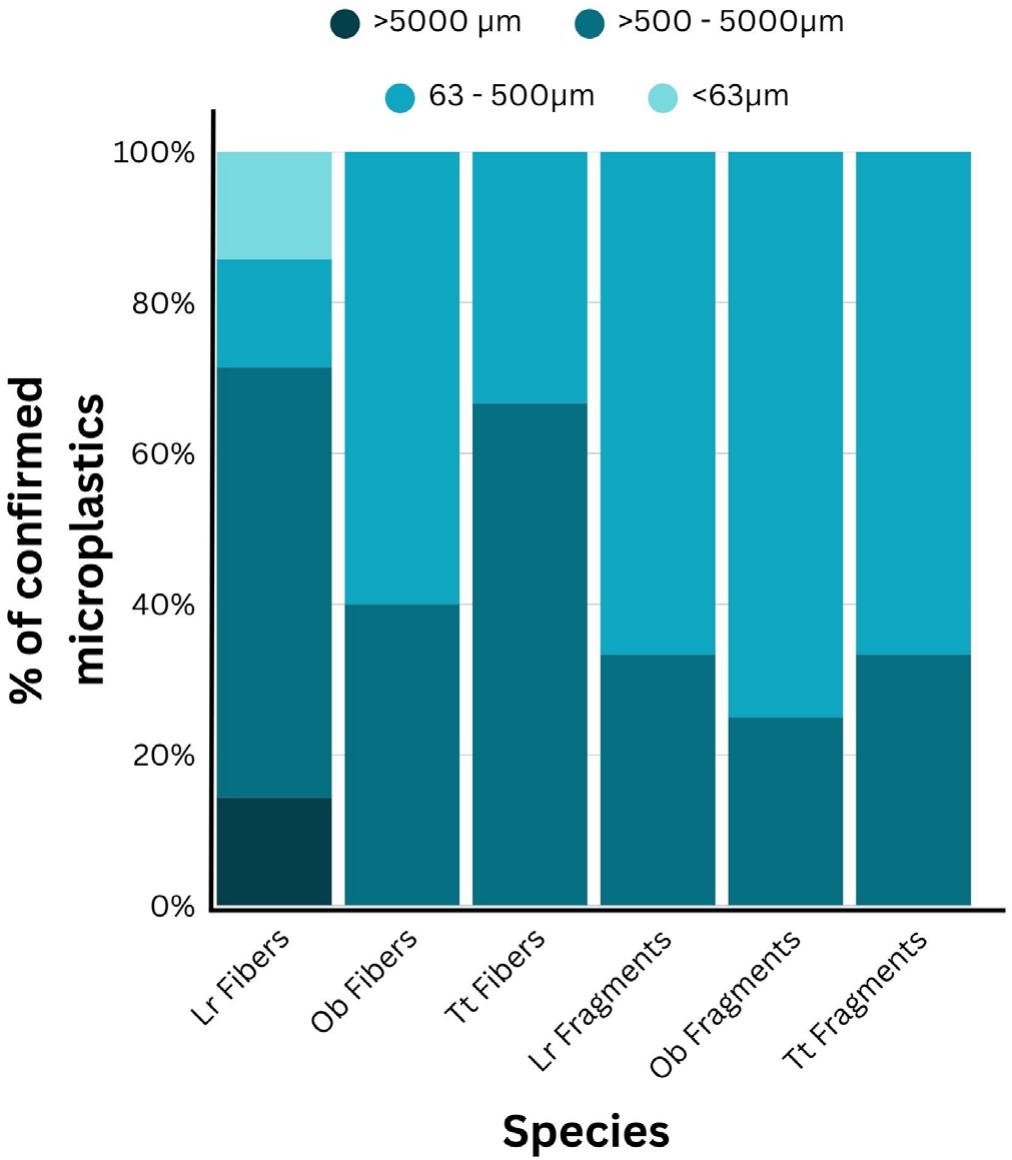
Lengths of confirmed microplastic fibers and combined fragments (films, foams, irregular shards, and tire wear fragments) from Raman subset of pinfish (Lr), Gulf toadfish (Ob), and dolphin (Tt) particles. Size category cutoffs are according to the accepted size range for microplastic particles (1–5000 μm) and the sieve sizes used in this study (63–500 μm).

All 34 confirmed microplastics were measured and organized into size categories based on length (Figure 6). All but one of the particles were 63 μm or longer, and the maximum length recorded was a fiber found in pinfish at 7346.4 μm; the only particle to exceed 5 mm in length (Figure 6, Table S2). The next largest particle was a fragment found in pinfish at 3382.1 μm. Overall, fibers fell more frequently into the largest size category, while fragments fell primarily within the 63–500 μm range (Figure 6). The fibers and fragments found in dolphin samples reflected the size trends in fish particles; more of these fibers were longer than 500 μm, while more of these fragments were 500 μm or less. Particles in pinfish samples (especially fibers) had the most size diversity, containing the longest and shortest particles in the subset (Figure 6, Table S2). The average length of all Raman subset particles for each species was as follows: 1709.3 μm for pinfish, 616.4 μm for Gulf toadfish, and 549.5 μm for dolphins.

### 3.4 Daily Particle Consumption in Dolphins From Pinfish and Gulf Toadfish

Using the particle abundance found in pinfish (mean 3.1 particles/g tissue; Table 3) and Gulf toadfish (mean 1.1 particles/g tissue; Table 3) samples, an estimate of the daily particle consumption among adult dolphins was calculated. We estimated that an adult dolphin in Sarasota Bay may ingest approximately 10,000 particles/day from these two fish species alone, adding up to at least 3.65 million particles each year for the largest males.

## 4 Discussion

This study found evidence of microplastic contamination in samples collected from bottlenose dolphins, pinfish, and Gulf toadfish. Microplastics were detected in bottlenose dolphin feces and gastric fluid, indicating that these dolphins are exposed to microplastics via ingestion. This is the first study to utilize both gastric and fecal samples from free‐ranging bottlenose dolphins to evaluate the potential for microplastic trophic transfer, providing critical insights into the ecological implications of microplastic pollution and a potential health risk to these animals. Several similarities emerged among the microplastics found in all three species that suggest trophic transfer may be occurring, as established by Nelms et al. (2018). First, fibers were the dominant particle shape observed across all bottlenose dolphin and fish samples, and every particle color present in bottlenose dolphin samples was also found in at least one of the fish species. Second, the lengths of fibers and fragments in the Raman analysis subset were comparable between fish and bottlenose dolphins. Third, Raman particle analysis confirmed the overlap of several polymers between the fish species and bottlenose dolphins.

It is not surprising that fibers were the dominant particle shape in all three species, as many other marine microplastic studies have also observed fibers as the most abundant particle type in marine mammals (Battaglia et al. 2020; Courville et al. 2024; Hernandez‐Gonzalez et al. 2018; Merrill et al. 2023; Nelms et al. 2019; Onay et al. 2025), fish (Abbasi et al. 2018; Bessa et al. 2018; Ding et al. 2019; Pereira et al. 2023), and the marine environment (Barrows et al. 2018; Yu et al. 2018). This abundance of fibers may represent the predominant type of microplastic pollution in Sarasota Bay. Microfiber contamination in the marine environment mostly occurs due to shedding during production (Liu et al. 2021), regular wear (Carney Almroth et al. 2018; Liu et al. 2021) and domestic machine washing of synthetic clothing and textiles that causes microplastic fibers to enter the environment through wastewater (Cai et al. 2020; De Falco et al. 2019; Eamrat et al. 2025). In fact, Napper and Thompson (2016) found that up to 700,000 fibers could be released from one 6 kg wash load. Domestic washing and contaminated wastewater are likely sources of microplastic fiber contamination in Sarasota Bay, as this marine environment is very close to an urban center. Additionally, although the majority of the microfibers contaminating the marine environment are synthetic, some are from natural materials such as cotton. In addition to plastic fibers, our Raman spectroscopy analysis detected 16 cotton fibers (19.5%) across all three species. The predominance of fibers in bottlenose dolphin samples contrasts with previous findings from Hart et al. (2022), where bottlenose dolphin samples primarily contained white foams and transparent films. This discrepancy could be related to the city ordinance in Sarasota, Florida that banned polystyrene containers (i.e., cups and to‐go containers) to be distributed from city‐owned properties in 2020, 1 year after the samples for the Hart et al. (2022) study were collected. Since the bottlenose dolphin samples from this study were collected from 2022 through 2024, perhaps the shift toward fiber abundance is related to this recent policy change.

In addition to overlap in shape and color, we also found overlap in particle sizes. In all three species, the majority of confirmed microplastic fragments were between 63 and 500 μm in length. In bottlenose dolphins and pinfish, the majority of microplastic fibers were greater than 500 μm in length, and while the majority of fibers found in toadfish fell into the 63–500 μm range, there were several that also exceeded 500 μm in length. The average length of the Raman subset particles in all three species fell between 500 and 1800 μm, which aligns with other studies on microplastics in marine mammals and fish. Ugwu et al. (2021) reviewed 132 studies on microplastic ingestion in marine invertebrates, largely including marine fish and mammals, and found that 73.6% of those studies had main particle sizes less than 2 mm (2000 μm). Additionally, the size of microplastics plays a role in how long particles are retained within their digestive systems. Roch et al. (2021) found a strong negative correlation between particle size and retention time for fish with stomachs, indicating that larger particles are egested quickly while smaller particles stay in the stomach for longer periods of time. Pinfish had a larger average particle size (1709.3 μm) than Gulf toadfish (616.4 μm) but had a particle load that was three times greater than toadfish. Finding larger quantities and sizes of particles in pinfish despite the tendency for bigger particles to be egested more quickly may indicate that pinfish experience more frequent microplastic exposure than Gulf toadfish. Pinfish also had a larger range of particle sizes, polymers, and particle colors than toadfish. As omnivores, larger adult pinfish consume seagrasses along with other small organisms like fish and crustaceans (Luczkovich 1988). The higher particle abundance and particle characteristic diversity in pinfish could be linked to their more diverse diet. Goss et al. (2018) found that epibionts can encrust microplastic particles onto seagrass blades, providing an additional ingestion pathway for pinfish that feed on seagrasses.

Lastly, all polymers found in fish were also found in bottlenose dolphin samples, which included PL, PP, PAM, and PET. PL, PP, and PET have been commonly found in other studies of marine fish (Pereira et al. 2023; Pingki et al. 2025; Sánchez‐Hernández et al. 2021) and mammals (Merrill et al. 2023; Nelms et al. 2019; Onay et al. 2025), while PAM is more uncommon. In the marine environment, PP and polyethylene (PE) are common in addition to polyamide (PA; Kannankai et al. 2022) and PET (Tang et al. 2023). PL and PET are commonly used to make synthetic clothing and textiles. PP and PE are used to make various mass‐produced plastic items, such as packaging, bottles, toys, etc. (Mohamadi 2023). PAM is used to make hydrogels (Simão et al. 2025), thicken liquids (Briscoe et al. 1999), or as a flocculant during water treatment processes (Shatat et al. 2019). Since Sarasota Bay is an estuary adjacent to an urban center, it is possible that some of these polymers are introduced into this environment through local wastewater and wastewater treatment processes. Additionally, PP and PE are used to make fishing nets and other fishing gear. Sarasota Bay is a popular spot for boating and other recreational water activities like fishing, so these polymers may have come from deteriorating fishing gear. Some polymer types were found in particles from dolphin samples but not in particles from fish samples (PE, polystyrene (PS), and polyurethane (PU)), which could have come from accidental ingestion or other fish species not included in this study. At least nine other prey fish species are known to be contaminated with microplastics (Conger et al. 2024) and hunted by Sarasota bottlenose dolphins (Barros and Wells 1998; Berens McCabe et al. 2010; Wells et al. 2013).

Our estimate for the daily particle consumption among adult dolphins in Sarasota Bay revealed that they may ingest approximately 10,000 particles every day from their typical consumption of pinfish and Gulf toadfish. Because our results provide evidence supporting the possibility of prey fish acting as vectors of microplastic contamination, they hold important implications for dolphins and humans. These results may raise even greater concern if they can be extended to other common prey species of Sarasota dolphins, which have also been shown to contain microplastics (Conger et al. 2024).

### 4.1 Strengths and Limitations

One limitation encountered in this study was the variability in sample volumes obtained from bottlenose dolphins during health assessments. Gastric and fecal samples were collected opportunistically, and in some cases, only trace amounts of the sample were recovered using filtered water. Because complete gastric or fecal contents were not obtained, standardized particle concentrations could not be reliably determined. Consequently, our results likely represent underestimates of microplastic ingestion in bottlenose dolphins. It is also worth noting that our reported particle counts likely underestimate the true number of particles in a sample, as blank‐correction eliminated some particles from our final results. For example, transparent and white fibers were the most common colors observed in blanks and, as a result, were most frequently eliminated from the final results. The goal of this study was not to quantify microplastic exposure in bottlenose dolphins, but rather to evaluate the overlap with prey in particle characteristics to better understand trophic transfer potential.

Secondly, while Raman spectroscopy is considered a state‐of‐the‐art method for confirming MPs in environmental and biological matrices and determining polymer composition (Jin et al. 2022), 35.7% of screened particles yielded inconclusive results. Similarly, Ertel et al. (2023) analyzed a subset of their particles using Raman spectroscopy and found that 15.4% were natural, 75% were anthropogenic, and 9.6% of their particles were inconclusive. The majority of the inconclusive particles were fibers. The small size of fibers can make it more difficult to get a proper spectrum without background contamination as opposed to broader fragment shapes (Silva et al. 2024). Additionally, colorants present in microplastic particles can mask peaks that would match those particles to known polymers, thus interfering with conclusive results (Azari et al. 2024; Silva et al. 2024). However, despite these challenges, the majority (64.3%) of the particle subset for Raman analysis was successfully matched to known materials, over half of which matched to synthetic polymers and confirmed the presence of plastic polymers in all three of the species analyzed. In addition to our own confident polymer matches, several of our most common polymers aligned with other studies that reported PP (Kannankai et al. 2022; Tang et al. 2023), PET (Tang et al. 2023), and PE (Kannankai et al. 2022; Tang et al. 2023) to be the most common polymers present in the marine environment.

Finally, for ethical, logistical, and legal reasons, we did not feed Sarasota bottlenose dolphins fish that were certain to be contaminated with microplastics to observe trophic transfer directly, so a direct causal relationship between microplastic contamination in the dolphins and prey fish could not be determined. Given the challenges of studying trophic transfer in controlled environments, researchers have also monitored specific marine areas over time to assess exposure trends. For example, Dromard et al. (2018) analyzed wild organisms across multiple trophic levels in a mangrove ecosystem for chlordecone, a toxic, persistent pesticide. They found that chlordecone concentrations increased with trophic level, demonstrating bioaccumulation as predators consumed contaminated prey. This approach is more commonly used in studies examining trophic transfer of microplastics, where contamination at lower trophic levels poses a consistent risk of pollutant introduction to higher predators. Nelms et al. (2018) expanded microplastic trophic transfer research further by comparing similarities in microplastic characteristics between the species to provide additional evidence supporting the potential for trophic transfer. We took a similar course in this study, observing microplastic exposure in all three species and highlighting overlapping microplastic characteristics. Additionally, we sampled fish that are known to make up large portions of the dolphins' diets (Berens McCabe et al. 2010; Wells et al. 2013) directly from the bottlenose dolphins' known ranges, and the sampling period of all three species overlaps (sampled during 2022–2024). While we did not directly observe MP consumption by Sarasota dolphins, this study found substantial evidence of overlapping particle characteristics between species that support the notion that prey fish are likely vectors of microplastic exposure.

## 5 Conclusion

Our results clearly indicate predominant microplastic contamination present in Sarasota Bay. Although we were not able to causally demonstrate microplastic trophic transfer between the bottlenose dolphins and prey fish studied, the extensive overlap observed in particle shapes, colors, sizes, and polymers across species provides researchers with reason to suspect that trophic transfer may be occurring in the Sarasota Bay ecosystem. This study highlights the need for further research on how microplastics travel through marine food webs and the health implications of microplastic ingestion and trophic transfer for marine wildlife.

## Author Contributions

**John E. Weinstein:** methodology, resources, writing – review and editing. **Randall S. Wells:** data curation, funding acquisition, investigation, project administration, resources, writing – review and editing. **Eric Conger:** investigation, data curation, writing – review and editing. **Elizabeth J. Berens McCabe:** investigation, project administration, resources, writing – review and editing. **Estella Martin:** conceptualization, data curation, formal analysis, investigation, methodology, validation, visualization, writing – original draft, writing – review and editing. **Tita Curtin:** investigation, data curation, writing – review and editing. **Millie A. Knowles:** investigation, writing – review and editing, data curation. **Robyn Faulkner Allen:** investigation, project administration, writing – review and editing. **Leslie B. Hart:** conceptualization, data curation, formal analysis, funding acquisition, investigation, methodology, project administration, resources, software, supervision, visualization, writing – original draft, writing – review and editing. **Christina N. Toms:** investigation, writing – review and editing, project administration. **Ayushi Gaur:** investigation, writing – review and editing, data curation. **Savannah M. Case:** data curation, investigation, writing – review and editing. **Miranda K. Dziobak:** investigation, validation, writing – review and editing.

## Funding

This research was supported by the National Institute of Environmental Health Sciences of the National Institutes of Health under Award Number R15ES034169. The content is solely the responsibility of the authors and does not necessarily represent the official views of the National Institutes of Health. Support for travel was provided by the School of Health Sciences at the College of Charleston. The Charles and Margery Barancik Foundation provided primary support to the Sarasota Dolphin Research Program (SDRP) for fish sampling operations in Sarasota Bay. Dolphin Quest Inc. provided primary support for dolphin health assessments.

## Conflicts of Interest

The authors declare no conflicts of interest.

## Supporting information


**Table S1:** Fish collection details.

**Table S2:** Raman particle subset results.

## Data Availability

The datasets generated and analyzed for this study can be found in the DRYAD data repository (DOI: 10.5061/dryad.4j0zpc8pw).
