# Supplementary material for: Microplastic Munchies: Exploring Microplastic Trophic Transfer Potential Between Two Key Prey Fish Species and Resident Common Bottlenose Dolphins ( Tursiops truncatus ) in Sarasota Bay, Florida
Source: Mar Mamm Sci. 2026 May 15;42(3):e70198. doi: 10.1111/mms.70198 (PMC13261691; doi:10.1111/mms.70198)
Supplement: Supplementary file 1 — Table S1: Fish collection details. [file MMS-42-e70198-s002.docx]

Table S1 – Fish Collection Details

| **Fish ID** | **Whole Fish Mass (g)** | **Latitude** | **Longitude** |
| --- | --- | --- | --- |
| *Lagodon rhomboides* | | | |
| 1 | 65.0 | 27.31178 | -82.56685 |
| 2 | 67.6 | 27.31178 | -82.56685 |
| 4_16862 | 121.2 | 27.31178 | -82.56685 |
| 4_19004 | 102.4 | 27.31513 | -82.54724 |
| 5_16862 | 126.6 | 27.31178 | -82.56685 |
| 5_19004 | 94.8 | 27.31513 | -82.54724 |
| 6 | 98.3 | 27.31513 | -82.54724 |
| 7 | 82.0 | 27.31513 | -82.54724 |
| 8 | 83.8 | 27.31513 | -82.54724 |
| 9 | 114.4 | 27.31513 | -82.54724 |
| 10 | 97.7 | 27.31513 | -82.54724 |
| 12 | 151.8 | 27.31513 | -82.54724 |
| 13 | 30.4 | 27.31513 | -82.54724 |
| 122 | 88.7 | 27.43263 | -82.65185 |
| 123 | 74.3 | 27.43263 | -82.65185 |
| 124 | 167.7 | 27.43263 | -82.65185 |
| 125 | 82.3 | 27.43263 | -82.65185 |
| 127 | 66.0 | 27.43263 | -82.65185 |
| 128 | 42.2 | 27.43263 | -82.65185 |
| 129 | 119.0 | 27.4876 | -82.69388 |
| 130 | 50.5 | 27.45666 | -82.68128 |
| 135 | 59.0 | 27.38772 | -82.56895 |
| 136 | 56.6 | 27.38772 | -82.56895 |
| 137 | 77.3 | 27.38772 | -82.56895 |
| 241 | 97.1 | 27.4536 | -82.6653 |
| 242 | 85.7 | 27.4536 | -82.6653 |
| 243 | 113.7 | 27.4536 | -82.6653 |
| 244 | 113.7 | 27.4536 | -82.6653 |
| 245 | 180.7 | 27.4536 | -82.6653 |
| 246 | 163.9 | 27.4536 | -82.6653 |
| 247 | 96.5 | 27.4536 | -82.6653 |
| 291 | 100.3 | 27.43599 | -82.64028 |
| 292 | 123.5 | 27.43599 | -82.64028 |
| 294 | 83.0 | 27.43599 | -82.64028 |
| 298 | 112.0 | 27.43599 | -82.64028 |
| *Opsanus beta* | | | |
| 17 | 61.1 | 27.42136 | -82.64988 |
| 18 | 37.0 | 27.42136 | -82.64988 |
| 19 | 62.8 | 27.42136 | -82.64988 |
| 20 | 46.4 | 27.42136 | -82.64988 |
| 21 | 50.1 | 27.42136 | -82.64988 |
| 29 | 80.2 | 27.47557 | -82.65622 |
| 134 | 70.2 | 27.38772 | -82.56895 |
| 188 | 48.6 | 27.42659 | -82.64182 |
| 189 | 90.7 | 27.42659 | -82.64182 |
| 202 | 104.0 | 27.47557 | -82.65622 |
| 212 | 108.8 | 27.48228 | -82.67213 |
| 213 | 120.6 | 27.48228 | -82.67213 |
| 248 | 49.0 | 27.4536 | -82.6653 |
| 259 | 267.5 | 27.50685 | -82.6923 |
| 271 | 55.1 | 27.39295 | -82.6241 |
| 272 | 52.6 | 27.39295 | -82.6241 |
| 279 | 683.2 | 27.34518 | -82.57757 |
| 305 | 123.8 | 27.41627 | -82.63256 |
| 369 | 48.3 | 27.42283 | -82.65201 |
| 375 | 104.7 | 27.42283 | -82.65201 |
| 376 | 67.1 | 27.31513 | -82.5472 |
| 380 | 99.6 | 27.40216 | -82.64118 |
| 381 | 88.8 | 27.40216 | -82.64118 |
| 409 | 47.2 | 27.52286 | -82.67765 |
| 410 | 61.8 | 27.52286 | -82.67765 |
| 411 | 76.0 | 27.52286 | -82.67765 |
| 412 | 80.6 | 27.52286 | -82.67765 |
| 418 | 538.2 | 27.52257 | -82.70396 |
| 424 | 173.1 | 27.39712 | -82.61616 |
| 425 | 83.2 | 27.39712 | -82.61616 |
