# Supplementary material for: Microplastic Munchies: Exploring Microplastic Trophic Transfer Potential Between Two Key Prey Fish Species and Resident Common Bottlenose Dolphins ( Tursiops truncatus ) in Sarasota Bay, Florida
Source: Mar Mamm Sci. 2026 May 15;42(3):e70198. doi: 10.1111/mms.70198 (PMC13261691; doi:10.1111/mms.70198)
Supplement: Supplementary file 2 — Table S2: Raman particle subset results. [file MMS-42-e70198-s001.docx]

Table S2 - Raman particle subset results

*Polymer abbreviations: PAM = polyacrylamide, PE = polyethylene, PET = polyethylene terephthalate, PL = polyester, PP = polypropylene, PS = polystyrene, PU = polyurethane*

| **Sample Species** | **Particle Shape** | **Color** | **Polymer** | **Dimensions**  **Min-Max (𝜇m)** | **Match Value (r)** |
| --- | --- | --- | --- | --- | --- |
| **Pinfish**  **(n=10)** | Fiber  Fiber  Fiber  Fiber  Fiber  Fiber  Fiber  Fragment  Fragment  Fragment | Black  Black  Black  Black  Black  Green  Black  Blue  Blue  Transparent | PAM  PL PL PL PL PL PET PP PP PP | 34.6 x 40.2  7.4 x 1517.7  15.7 x 743.9  9.1 x 2402.2  29.6 x 203.1  11.4 x 7346.4  18.7 x 880.1  85.6 x 253.9  168.7 x 323.6  461.8 x 3382.1 | 0.72  0.70  0.88  0.73  0.81  0.79  0.89  0.91  0.90  0.70 |
| **Gulf Toadfish**  **(n=9)** | Fiber  Fiber  Fiber  Fiber  Fiber  Fragment  Fragment  Fragment  Fragment | Black  Black  Green  Green  Black  Transparent  White  White  White | PL  PL PL PET PP PET PET  PET PET | 17.0 x 1161.3  24.0 x 1236.8  25.4 x 385.6  16.6 x 485.7  19.4 x 434.6  989.7 x 1544.5  80.5 x 109.1  57.1 x 92.5  90.6 x 97.8 | 0.74  0.81  0.78  0.89  0.75  0.92  0.95  0.94  0.74 |
| **Dolphin**  **(n=15)** | Fiber  Fiber  Fiber  Fragment  Fragment  Fragment  Fragment  Fragment  Fragment  Fragment  Fragment  Fragment  Fragment  Fragment  Fragment | Blue  Transparent  Green  Transparent  Green  Black  Blue  Blue  Transparent  Blue  Blue  Blue  Blue  Grey  Transparent | PL PE PP PAM PAM  PE  PE PE PET PET PP PP PS PS PU | 15.1 x 1184.8  28.7 x 1542.8  23.9 x 233.4  58.2 x 1243.1  77.6 x 138.4  84.6 x 188.8  164.5 x 292.8  253.6 x 538.9  321.9 x 451.7  91.7 x 280.5  156.8 x 432.8  198.7 x 317.5  63.2 x 238.4  333.3 x 619.4  430.4 x 539.6 | 0.85  0.80  0.70  0.72  0.84  0.89  0.87  0.71  0.73  0.89  0.85  0.88  0.89  0.85  0.90 |
